# Dr. Frederick W. Smith

**Published:** 1917-09

**Authors:** 


**Dr. Frederick W. Smith,** Syracuse 1908, of Syracuse, died July 31, aged 59.

